# Improving self-directed learning ability of medical students using the blended teaching method: a quasi-experimental study

**DOI:** 10.1186/s12909-023-04565-x

**Published:** 2023-08-29

**Authors:** Si Ying Lu, Xiang Peng Ren, Huang Xu, Dong Han

**Affiliations:** 1 Department of Clinical Medicine, Jiaxing University Medical College, Jiaxing, 314001 Zhejiang China; 2 Department of Biochemistry and Molecular Biology, Jiaxing University Medical College, Jiaxing, 314001 Zhejiang China

**Keywords:** Biochemistry, Teaching methods, Self-directed learning

## Abstract

**Background:**

Self-directed learning (SDL) is one of the most important abilities for medical students in terms of their future clinical medical practice. During the blended teaching process, teachers can design a variety of learning activities to cultivate students’ SDL abilities. This study aimed to assess the differences between the SDL abilities of medical students using blended and traditional didactic teaching.

**Methods:**

This study included 239 medical students from eight administrative classes. The students were divided into two groups: (1) the experimental group (EG), which included 119 students from four administrative classes, and (2) the control group (CG), which included 120 students from the remaining four classes. From February to July 2022, blended teaching methods were applied to the EG group, and SDL abilities were assessed in comparison to the CG group receiving traditional didactic teaching methods.

**Results:**

At the end of the semester, significant differences (p < 0.05) were observed between EG and CG in all six SDL ability factors. Furthermore, when k-means cluster analysis was used to analyze the learning behavior of students in the EG after classifying them as comprehensive, interactive, and passive types, significant differences were observed in all six Self-directed learning factors of students with the comprehensive type, whereas significant differences were observed in four factors (setting learning goals and plans, self-monitoring and regulation, information processing, and communication and cooperation) of students with the interactive type. For students with passive type, only one factor of SDL (information processing) showed significant improvement. There were on differences between comprehensive, interactive, and passive types of CG.

**Conclusion:**

The blended teaching approach is better than the conventional didactic teaching for cultivating clinical medical students’ SDL abilities.

## Background

The self-directed learning (SDL) ability of medical students plays a vital role in the development of medical education [1, 2]. Medicine is a discipline closely related to life safety and health benefits, and hence medical students are required to be able to update their knowledge quickly and remain engaged in lifelong learning [3, 4]. For medical students, SDL ability is the foundation for lifelong learning [5, 6]. Therefore, one of the important tasks of higher medical education is to develop SDL abilities in students.

SDL is the process of students to constantly monitor and adjust their cognitive state, observe and apply various learning strategies, adjust their learning behaviors, and make efforts to create and use physical and social resources that facilitate learning [7–9]. Furthermore, SDL ability is considered the ability to learn while actively realizing the responsibility of learning [10, 11]. In the blended teaching process, teachers allow students to design teaching materials for course delivery, stimulating students’ SDL abilities [12, 13]. Notably, to cultivate the SDL ability of students, educational researchers have also explored this. Padugupati et al. found that a blended learning environment was a dynamic learning space that could effectively improve students’ learning behavior, including SDL ability [14]. A blended learning approach involving Indian medical students found that curriculum design should improve learning activities to promote SDL ability [15]. Developing a favorable education and learning environment by using modern educational technologies is prioritized in many universities to improve the SDL abilities of medical students.

A number of methods are available to assess the SDL ability of students, including the survey method, the interview method, teacher evaluation, and behavior observation [16–18]. Since the SDL definition varies among different researchers, there are subtle differences in the indicators used to assess the SDL ability of medical students. Garrison proposed a comprehensive model for SDL ability where the SDL ability was divided into three dimensions: learning motivation, self-monitoring, and self-management [19]. Wang Xiaodan et al. developed an assessment scale to evaluate the SDL ability of medical students [20]. This scale consists of two sub-scales (self-motivated beliefs and objective behavior) and six factors (self-motivation, learning beliefs, setting learning goals and plans, self-monitoring and regulation, information processing, and communication and cooperation) among a total of 30 items. This assessment scale has been widely used to evaluate the SDL abilities of medical students.

In recent years, the educational plethora has witnessed significant changes, including the development of online teaching resources, the construction of smart classrooms, the introduction of smart teaching tools, and the implementation of flipped and blended teaching methods [21, 22]. The blended teaching method is an advanced teaching mode that uses the intelligent network teaching system (in the backdrop of the internet) to make courses more intelligent and refined. During the blended teaching process, SDL behavior data, such as micro-lecture learning, material downloads, and case discussions, are fully recorded through the course web platform and smart teaching tools.

Dina Adinda et al. pointed out that blended teaching is a good way to improve students’ self-directed learning ability, together with promoting self-directed learning ability through both face-to-face and online teaching [23]. The present study focused on two factors associated with self-directed learning ability, namely, competency in self-regulated and self-directed learning skills, and designed blended teaching activities for 2021 students majoring in clinical medicine at Jiaxing College. These included 64 sessions of a biochemistry course that involved both online and face-to-face instruction, and three stages of learning, namely, before, during, and after class.

The Biochemistry and Molecular Biology course at Jiaxing University is the first-level blended course recognized by the Ministry of Education of Zhejiang Province, China. In this course, student-centered blended teaching was performed for 3 years, with more than 500 students and more than 100,000 student visits per year. During different learning behaviors, such as micro-lecture learning and material downloading, an online teaching platform and intelligent classroom teaching tools can record learning behavior data comprehensively.

In this study, the blended teaching method was used to cultivate students’ SDL abilities. Smart teaching tools (Wisdom Tree classroom interactive tools) were used to collect students’ learning behaviors and to assess differences in students’ SDL ability before and after the blended teaching experiment conducted for a semester.

## Methods

### Participants

**Fig. 1:**
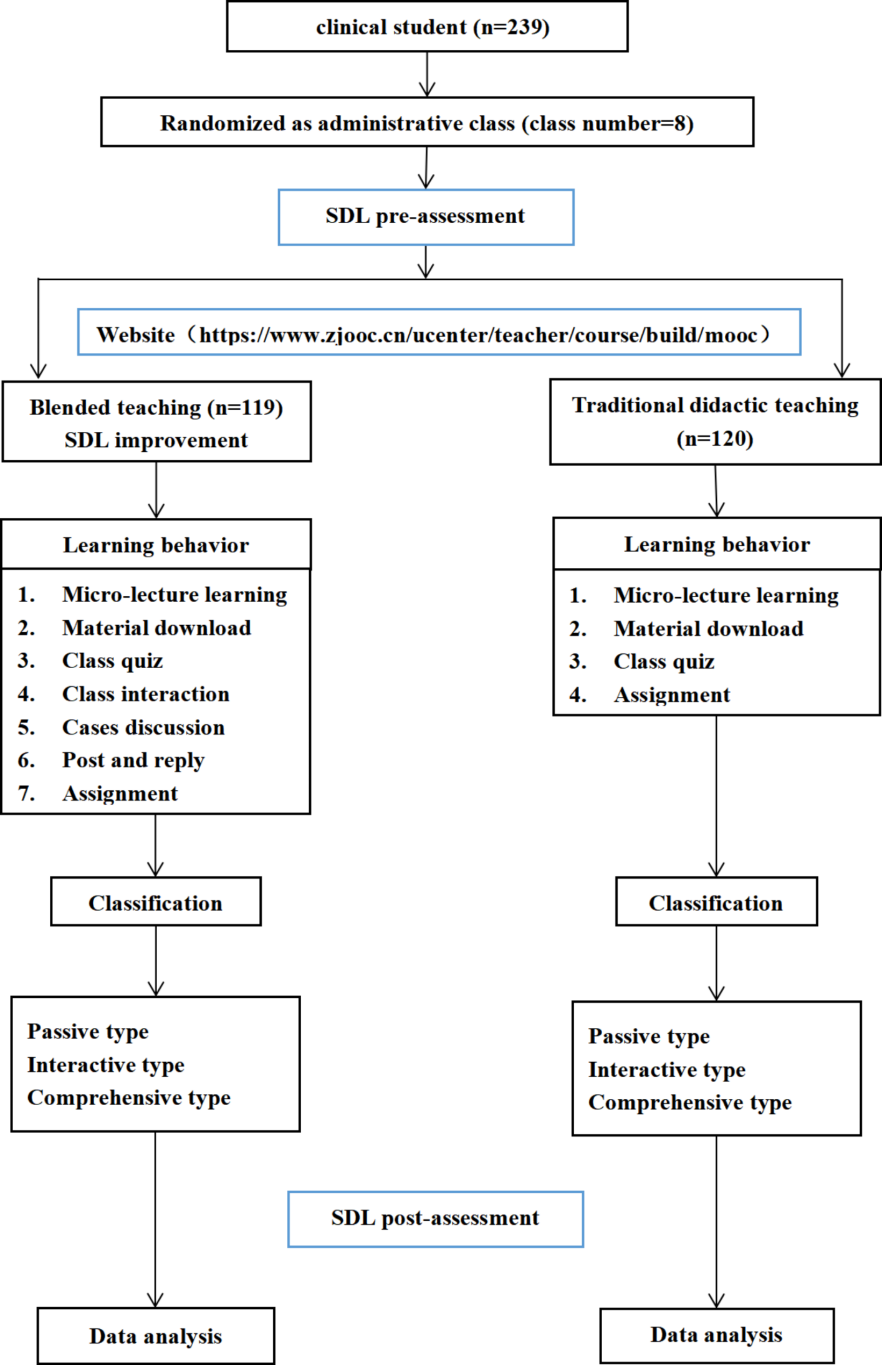
Flow chart showing teaching research design

This study included medical students (major in clinical medicine) of the Jiaxing University, the included students were divided into the following two groups: (1) experimental group (EG), containing 119 students from four administrative classes and (2) control group (CG), containing 120 students from four other administrative classes. Our research followed the guideline for reporting evidence-based practice educational interventions and teaching (GREET) statement which include systematic review, educational intervention, and testing [24–26] Eight administrative classes of the 2021 class in clinical medicine at Jiaxing University were randomly assigned numbers of 1–8. These numbers were written on small strips of paper of identical size, shape, and texture and crumpled into small balls, after which they were placed in an opaque box and mixed thoroughly. Four numbered sticks were drawn from the box without replacement and the numbers on them were recorded. The classes corresponding to the numbers were identified and used as the experimental group. The remainder of the classes served as the control group.The teaching design was shown in Fig. 1. Administrative class is the unit of student management of the Jiaxing University. It refers to a fixed group of students who share the same academic schedule in their teaching program. Students were assigned to either group based on the balance of age, gender, and scores obtained in the college entrance examination. Every administrative class has 28 to 31 students.

### Teaching activities in the CG and EG

In general, the participants of EG and CG were instructed through teaching models using course websites and digital resources. The participants of EG were told to use class interaction, case discussion and post and reply learning activities during the whole learning process. The participants of EG and CG accepted the blended teaching method and traditional didactic teaching method respectively.

Teaching activities in the CG were teacher-dominated in-class presentations. Students were encouraged to learn according to the teaching schedule, which included teacher-assigned pre-class preparation, in-class presentations, and after-class assignments. During this learning process, the types of learning behaviors of the EG were determined using the Zhejiang Open Course Sharing Platform (https://www.zjooc.cn/ucenter/teacher/course/build/mooc).

**Fig. 2:**
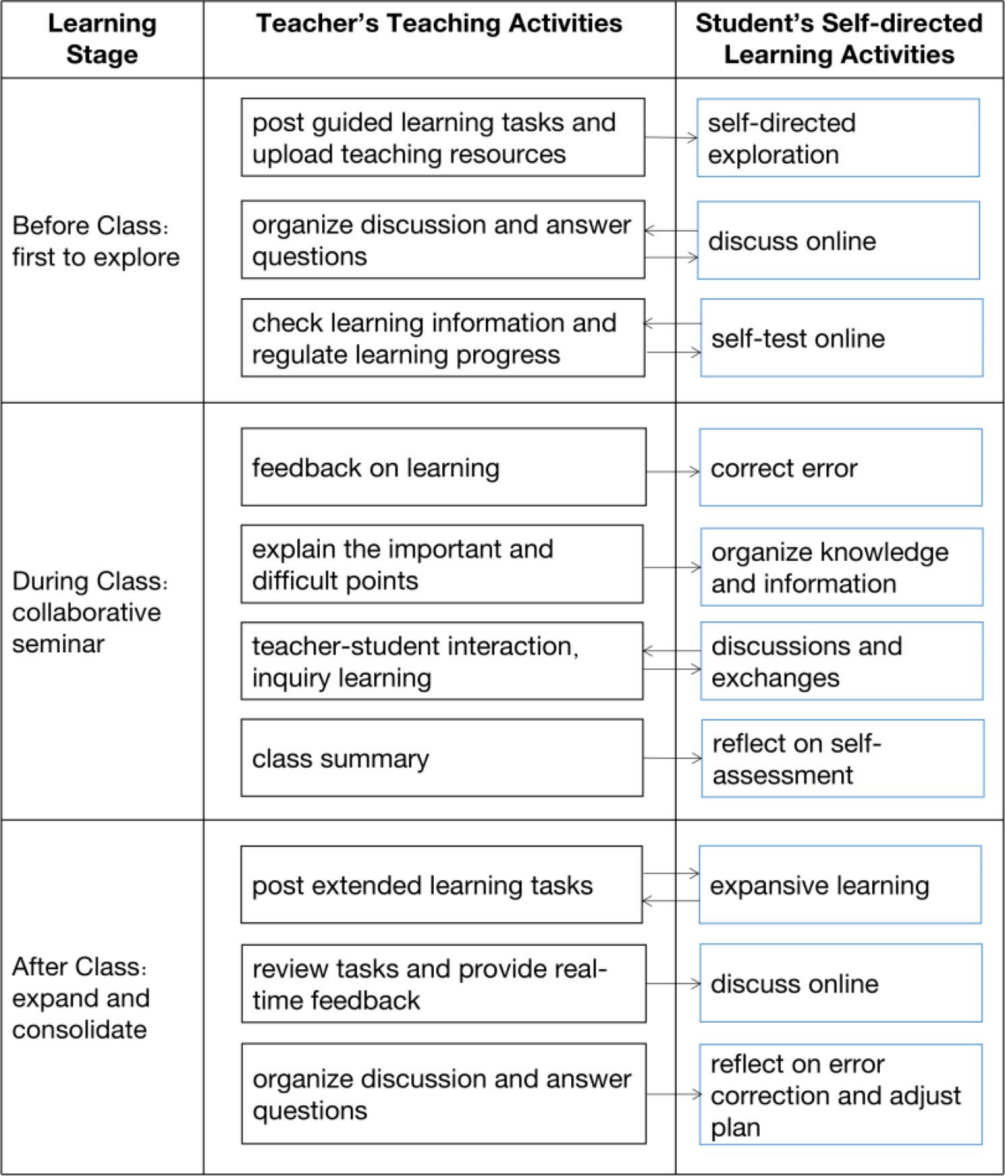
Flow chart showing teaching method used in the EG

In the EG, a blended-teaching method was applied (Fig. 2). Here, pre-class preparation, in-class presentations, and after-class assignments were organized using the blended-teaching philosophy. During this learning process, the types of learning behaviors of the EG were determined using the Zhejiang Open Course Sharing Platform (https://www.zjooc.cn/ucenter/teacher/course/build/mooc) and the Wisdom Tree flipped classroom tool. The flipped classroom from Wisdom Tree is an effective tool for teachers that automatically records teacher-student and student-student interactions of EG. Table 1 lists specific data recorded using this tool. The application of the two teaching network platforms comprehensively covered the whole teaching process before, during, and after classes.

**Table 1:** List of learning behaviors of the blended-teaching method

Teaching Session	Learning Behavior	Observable Indicator	Unit	Recording Mode
Before Class	Micro-lecture learning	Duration of micro-lecture learning	Minute	Zhejiang province open online course sharing platform
	Material download	Number of data downloads	Frequency	Zhejiang province open online course sharing platform
During Class	Class quiz	Score of class quiz	Percent	Zhejiang province open online course sharing platform
	Class interaction	Number of participations of class interaction	Frequency	Wisdom tree flipped class
	cases discussion	Number of participations of discussion of cases	Frequency	Wisdom tree flipped class
After Class	Post and reply	Number of posts and replies	Frequency	Zhejiang province open online course sharing platform
	Assignment	Number of homework completed	Frequency	Zhejiang province open online course sharing platform

### Assessment of the SDL ability

The SDL ability was assessed using the assessment scale developed by Wang Xiaodan et al. This scale consists of two sub-scales (self-motivated beliefs and objective behavior) and six factors (self-motivation, learning beliefs, setting learning goals and plans, self-monitoring and regulation, information processing, and communication and cooperation) among a total of 30 items. Furthermore, the Likert scale is a 5-point response scale, with a score of 5 − 1 representing “fully meets”, “generally meets”, “fair”, “not fully meets,“ and “not at all meets”. The scale of the six factors is based on a 5-point response system, with a 5 − 1 scale representing decreasing degrees of “fully conform”, “basically conform”, “generally conform”, “not conform”, and “not conform at all”, and a score range of 30−150. The Cronbach’s alpha coefficient for the original scale was 0.929, with a reliability of 0.992. The assessment of SDL ability was conducted between EG and CG before and after the semester.

### K-means clustering analysis

K-means is a classification algorithm that determines the attributes of research objects based on the similarity between different object features [27]. In this study, the k-means clustering algorithm was used to cluster then learners according to learning behavior data of the EG and CG, and the learners were divided into comprehensive, interactive, and passive types according to K = 3.

### Learning behavior data and statistical analysis

Learning behavior data analysis was performed using Z-scores and radar maps.The z-score was calculated as: z-score=(score of the behavior sequence-score of the behavior min)/(score of the behavior max-score of the behavior min).We compiled the factor scores of the SDL ability of CG and EG using an independent sample T-test. We also compared the factor scores of SDL ability by the CG and EG before and after that were generated after clustering using a paired sample T-test. The *x*^2^-test was used to compare the age, gender composition and entrance score between the two group. All statistical analysis was performed using SPSS V.27 for Windows. Age and scores for each factor of the SDL ability assessment scale were expressed as mean ± standard deviation, and P-values of less than 0.05 were considered significant.

## Results

### Participants

Table 2 shows the demographic data of the study participants. No significant differences were observed in the age and sex of participants between the two groups (*p* > 0.05).

**Table 2:** Demographic characteristics of study participants

Characteristics	Experimental group (n = 119)	Control group (n = 120)	Pvalue
Age (years)	19.47 ± 0.45	19.77 ± 0.52	*p* > 0.05
Sex			
Female	50.4%	53.3%	*p* > 0.05
Male	49.6%	46.7%	*p* > 0.05
Entrance score	579.84 ± 16.75	581.90 ± 16.68	*p* > 0.05

### Learning behavior classification of the EG and CG

**Fig. 3:**
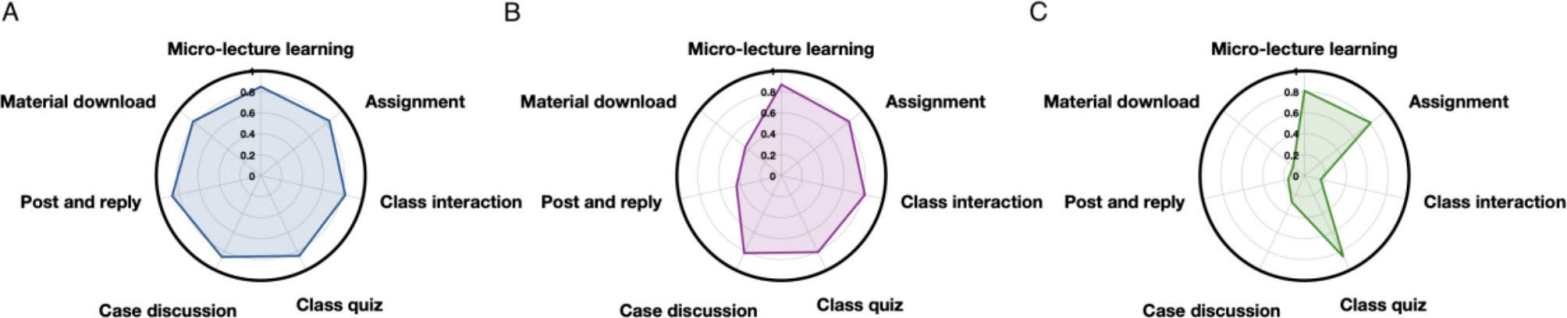
Learning behavior radar chart of EG

**Fig. 4:**
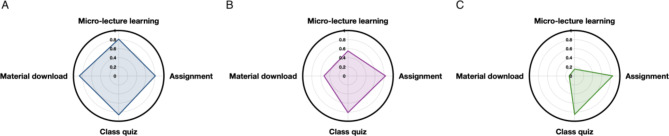
Learning behavior radar chart of CG

Based on the cluster analysis, students were classified into 3 categories, as shown in Figs. 3 and 4. The numbers of students were 41, 38, and 40 in the comprehensive, interactive, and passive types respectively of EG. Based on the cluster analysis, students were classified in the comprehensive, interactive, and passive types respectively of CG. The numbers of students were 44, 35, and 41 in the comprehensive, interactive, and passive types respectively of CG.

We prepared a 7-point radar chart based on the mean value of the z-score of the learning behavior data of students in the EG in the three categories. As shown in Fig. 3, the mean values of the z-score in the comprehensive category were all above 0.8 (Fig. 3A), whereas material download, post and reply these values were lower than 0.4 in the interactive type category (Fig. 3B). Furthermore, the passive type category demonstrated very low mean values of z-score in the majority of classifications except micro-lecture learning, assignment, class quiz where the value was above 0.8 (Fig. 3C).

We also prepared a 4-point radar chart based on the mean value of the z-score of the learning behavior data of students in the CG in the three categories. As shown in Fig. 4, the mean values of the z-score in the comprehensive category were all above 0.8 (Fig. 4A), whereas micro-lecture learning, material download behavior data values were lower than 0.6 in the interactive type category (Fig. 4B). Furthermore, the passive type category demonstrated very low mean values of z-score in the majority of classifications except micro-lecture learning and class quiz (Fig. 4C).

### SDL ability assessment

**Fig. 5:**
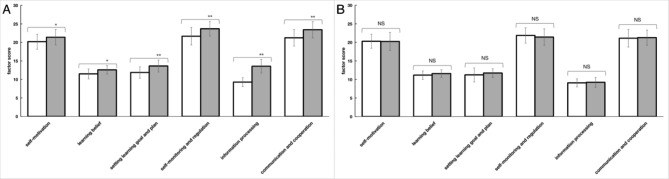
Assessment of SDL ability of EG and CG. Mean scores of all six factors SDL ability pre-experiment and post-experiment in EG (**A**). Mean scores of self-motivations, learning belief, setting learning goal and plan, self-monitoring and regulation, information processing and communication and cooperation all six factors pre-experiment and post-experiment in CG (**B**). Data are presented as the means ± standard deviation; * p < 0.05 ** p < 0.01 and NS: no significant difference

At the end of the semester, significant differences (p < 0.05) were observed between EG and CG in all six SDL ability factors (Fig. 5A). Furthermore, in comparison to CG, significant differences were observed in all six SDL factors of students with comprehensive type, whereas significant differences were observed in four factors (setting learning goals and plans, self-monitoring and regulation, information processing, and communication and cooperation) of students with interactive-type. For students with passive type, only one factor of SDL (information processing) showed significant improvement compared with CG (Fig. 5B).

**Fig. 6:**
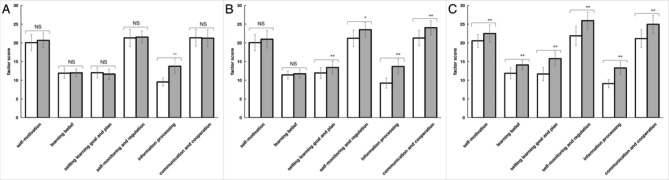
Assessment of SDL ability of comprehensive type, interactive-type and passive type category in EG. Mean scores of all six factors pre-experiment and post-experiment in comprehensive type of EG (N = 40, **A**). Mean scores of all six factors pre-experiment and post-experiment in interactive type of EG (N = 38, **B**). Mean scores of all six factors pre-experiment and post-experiment in passive type of EG (N = 41, **C**) Data are presented as the means ± standard deviation; * p < 0.05 ** p < 0.01 and NS: no significant difference

After classification, significant differences (p < 0.05) were observed between comprehensive type in EG in all six SDL ability factors (Fig. 6C). Furthermore, whereas significant differences were observed in four factors (setting learning goals and plans, self-monitoring and regulation, information processing, and communication and cooperation) of students with interactive-type in EG (Fig. 6B). For students with passive type in EG, only one factor of SDL (information processing) showed significant improvement compared with CG (Fig. 6A).

**Fig. 7:**
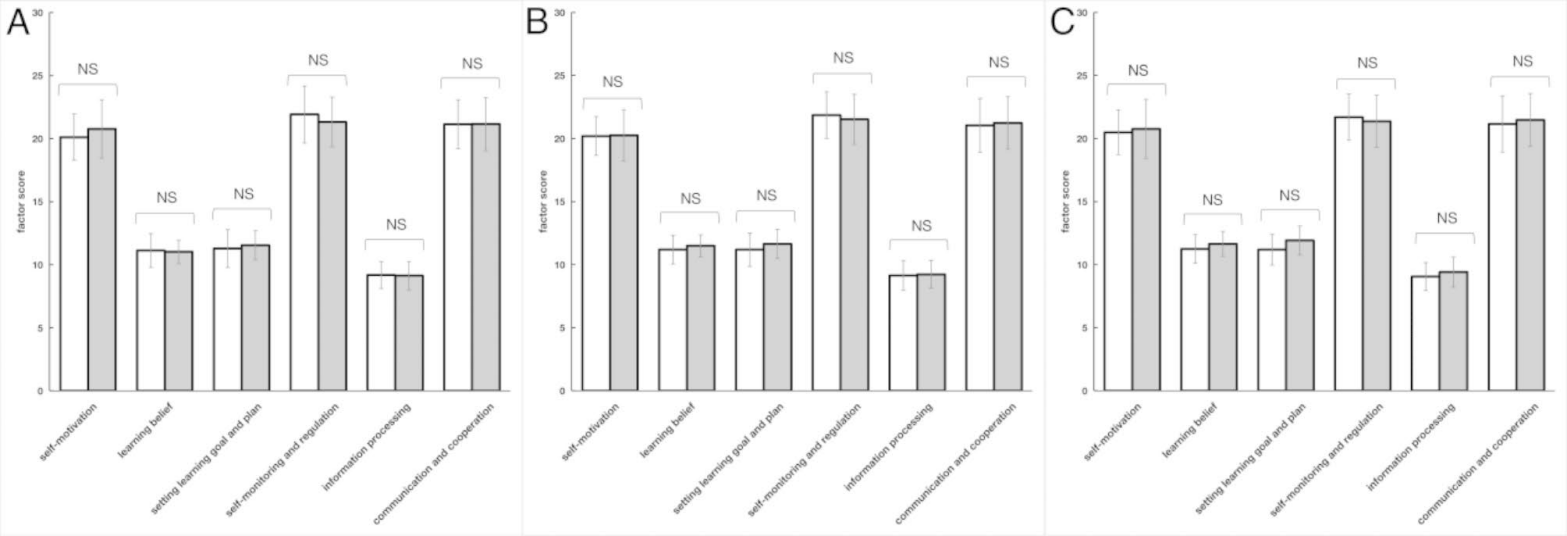
Assessment of SDL ability of comprehensive-type, interactive-type and passive-type category in CG. Mean scores of all six factors pre-experiment and post-experiment in passive type of CG (N = 41, **A**). Mean scores of all six factors pre-experiment and post-experiment in passive type of CG (N = 35, **B**) Mean scores of all six factors pre-experiment and post-experiment in passive type of CG (N = 44, **C**). Data are presented as the means ± standard deviation; * p < 0.05 ** p < 0.01 and NS: no significant difference

After classification, no significant differences (p>0.05) were observed between interactive-type in CG in all six SDL ability factors (Fig. 7A). Furthermore, no significant differences were observed in all six SDL factors of students with comprehensive type (Fig. 7B). For students with passive type, no significant differences (p>0.05) were observed showed significant between passive-type in CG (Fig. 7C).

## Discussion

### Blended teaching enhances students’ SDL ability

SDL is a process in which students consciously set learning objectives, choose learning methods, monitor the learning process, and assess learning outcomes [28, 29]. One of the goals of medical education is to cultivate medical students’ SDL ability, and an important task of medical personal training is to cultivate advanced medical talents who can acquire knowledge independently [30]. With the development of blended learning mode, teachers should combine teaching with learning resources, stimulate learners’ interests in learning, and create conditions for students to achieve exploratory learning [31]. During the blended teaching, teachers stimulate learning by designing activities before, during, and after classes. In this method, online tools are used to provide initial basic learning, while offline classroom teaching sessions are organized with a variety of activities and tasks to consolidate and enhance the knowledge [32]. The process of completing tasks is also the process of applying strategies to solve problems. The process of learning through micro-lecture learning, material download, class quizzes, case discussions, and questions after class is also the process of developing SDL ability.

In this study, blended teaching method was implemented for the development of SDL ability in medical students before, during, and after classes, and students are constantly prompted to self-examine, self-monitor, and self-adjust during class interactions, quizzes, case discussions, and other teaching sessions. When EG students were categorized further into categories, comprehensive type students demonstrated clearer motivation and learning goals during the blended teaching method, with improvements in all six dimensions of SDL ability. Further, interactive type students did not experience changes in two factors of SDL abilities including setting learning goals and plans and self-monitoring and regulation. For passive students, only the interactive ability has improved after blended teaching experiment. Further statistical research is needed to correlate the data on the learning behaviors of different types of learners with the improvement of the SDL ability.

### Teaching design improves students’ SDL ability

The development of SDL ability is a long-term process, and the key factors include motivation and monitoring [33].In this study, When CG students were categorized further into categories, comprehensive type students too, all six dimensions of SDL ability has no difference.

We also clustered the students into comprehensive, interactive, and passive types according to their learning behavior data, and there were no significant differences in SDL ability between these three categories of students in the CG. Students in the CG only followed the teacher’s lecture content to master the knowledge but did not apply thinking and discussion, which are precisely the learning behaviors that blended teaching is designed to address in contrast to traditional didactic teaching. The learning behaviors that made the difference between the SDL ability of the two groups were thinking and discussion. Learning from lectures or downloaded material from the instructional website with no learning purpose did not improve the SDL abilities of the students. Our research showed the effectiveness of cultivating SDL ability under the teachers’ systematic teaching design. The teacher’s systematic teaching design is also a crucial factor in the development of SDL ability.

The assessment of SDL ability includes several dimensions and factors, with the core of the evaluation mainly involving self-motivation and the ability to self-monitor and self-adjust. Self-motivation is key to SDL. Self-monitoring represents the responsibility to maintain a socially required level of clinical competence, and is also related to the responsibility of setting, achieving, and maintaining self-determined goals. Self-adjustment includes the ability to self-check, provide feedback, and control and regulate actions. It also refers to the students’ ability to regulate task-operating factors, such as learning objectives, tasks, materials, and methods [30]. To develop self-adjustment and self-monitoring qualities, teachers should provide encouragement and guidance to students that allow them to check, control, and regulate their own learning according to subjective psychological factors, such as learning interests and motivation.

We also clustered the students into comprehensive, interactive, and passive types according to their learning behavior data, and there were no significant differences between the SDL ability of these three categories of students in CG. Students of CG just followed the teacher’s lecture content to master the knowledge but lacked thinking and discussing, which are precisely the learning behaviors that designed in the blended teaching but not in the traditional didactic teaching. The learning behaviors that made the difference between the SDL ability of the two groups were thinking and discussion. The lecture learning and download learning behaviors of comprehensive students in CG which recorded by the instructional website without the learning purpose did not lead to the improvement of students’ SDL ability. Our research shows that the process of cultivating SDL ability is the process of students’ SDL ability culturing under the teachers’ systematic teaching design. The teacher’s systematic teaching design is also a very crucial factor in the process of developing SDL ability.

The assessment of SDL ability includes several dimensions and factors, and the core of the evaluation mainly includes self-motivation, self-monitoring ability, and self-adjustment ability. Self-motivation is the key to SDL. Self-monitoring is the responsibility to maintain a socially required level of clinical competence, and it is also related to the responsibility of setting, achieving, and maintaining self-determined goals. Self-adjustment includes the ability to self-check, provide feedbacks, and control and regulate the actions. It also refers to the students’ ability to regulate task-operating factors, such as learning objectives, tasks, materials, and methods [30]. In order to develop self-adjustment and self-monitoring qualities, teachers provide encouragement and guidance to students that allow them to check, control, and regulate their own learning according to subjective psychological factors, such as learning interests and motivation.

According to the present findings, the blended teaching method was used more popular during and after the covid-19. In their study, Chen M et al. stated that blended teaching can be made to address low student engagement and poor classroom participation during covid-19 [34]. In another study conducted during the COVID-19 pandemic, David O Obada et al. argued that blended online teaching and learning strategy was a new pedagogy for adapting classrooms in developing countries [35]. In a study conducted after this pandemic, Xue-Tao Fu et al. argued that blended teaching is beneficial to students’ learning and stimulates their enthusiasm, cultivates clinical thinking ability, and improves teaching quality [36]. The blended teaching method was used in medical education in developing countries such as China more and more deeply. Moreover, our study was just benefic research for the blended teaching method.

There is no unified approach for cultivating independent SDL ability. Different specialties, different courses, and different lecture subjects have their own methods of cultivating SDL ability. The cultivation of SDL ability not only requires teaching design but also a diverse and complex evaluation system to assess SDL ability. This preliminary study on medical students’ SDL ability using a blended teaching method lays the foundation for the cultivation of SDL ability in medicine discipline students.

## Conclusion

In conclusion, the blended teaching model may be effective for improving medical students’ SDL ability compared with the traditional didactic method. The blended teaching model effectively enhanced students’ SDL ability factors of self-motivation, learning beliefs, setting learning goals and plans, self-monitoring and regulation, information processing, and communication and cooperation.

## Data Availability

Data collection containing teaching data is available upon request from the corresponding author.
